# Meniscal Ossicle Mimicking a Radial Meniscal Tear

**DOI:** 10.5334/jbsr.2125

**Published:** 2020-06-26

**Authors:** Guillaume Vangrinsven, Filip Vanhoenacker

**Affiliations:** 1 AZ Sint-Maarten, BE; 2 University (Hospital) Antwerp/Ghent, BE

**Keywords:** meniscal ossicle, meniscal root, meniscal tear, MRI, radiography

## Abstract

**Teaching Point:** A meniscal ossicle may be misdiagnosed as a tear of the posterior horn of the medial meniscus or an intra-articular loose body.

## Case Presentation

**Figure 1:**
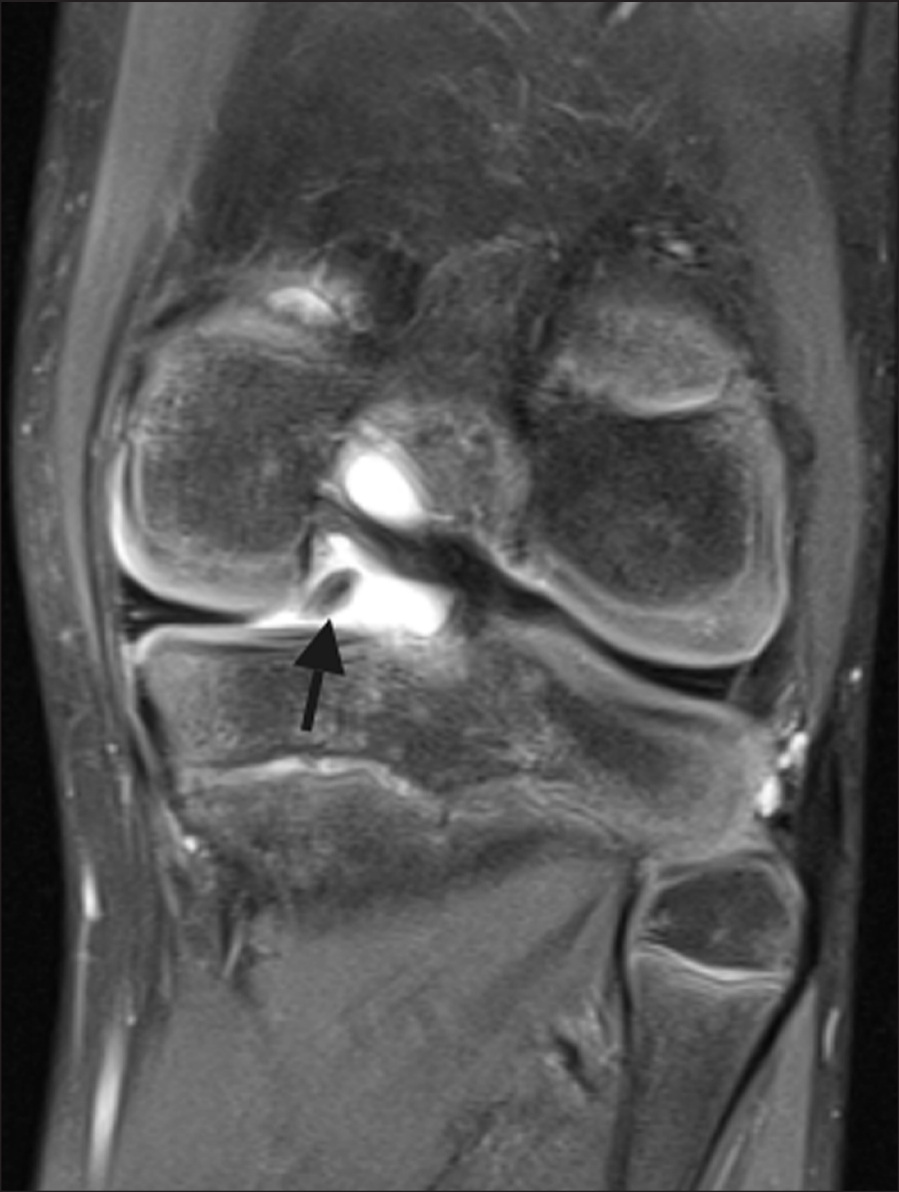


**Figure 2:**
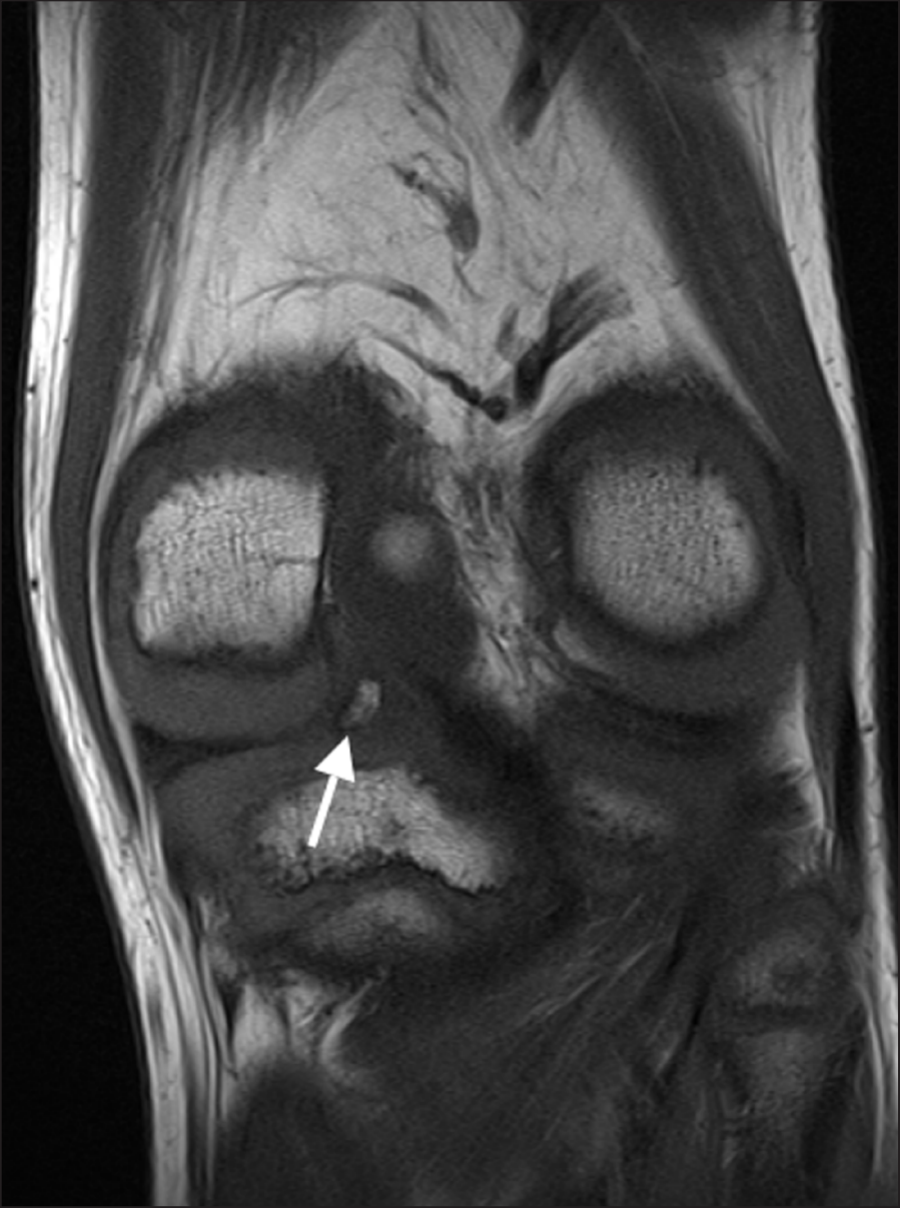


**Figure 3:**
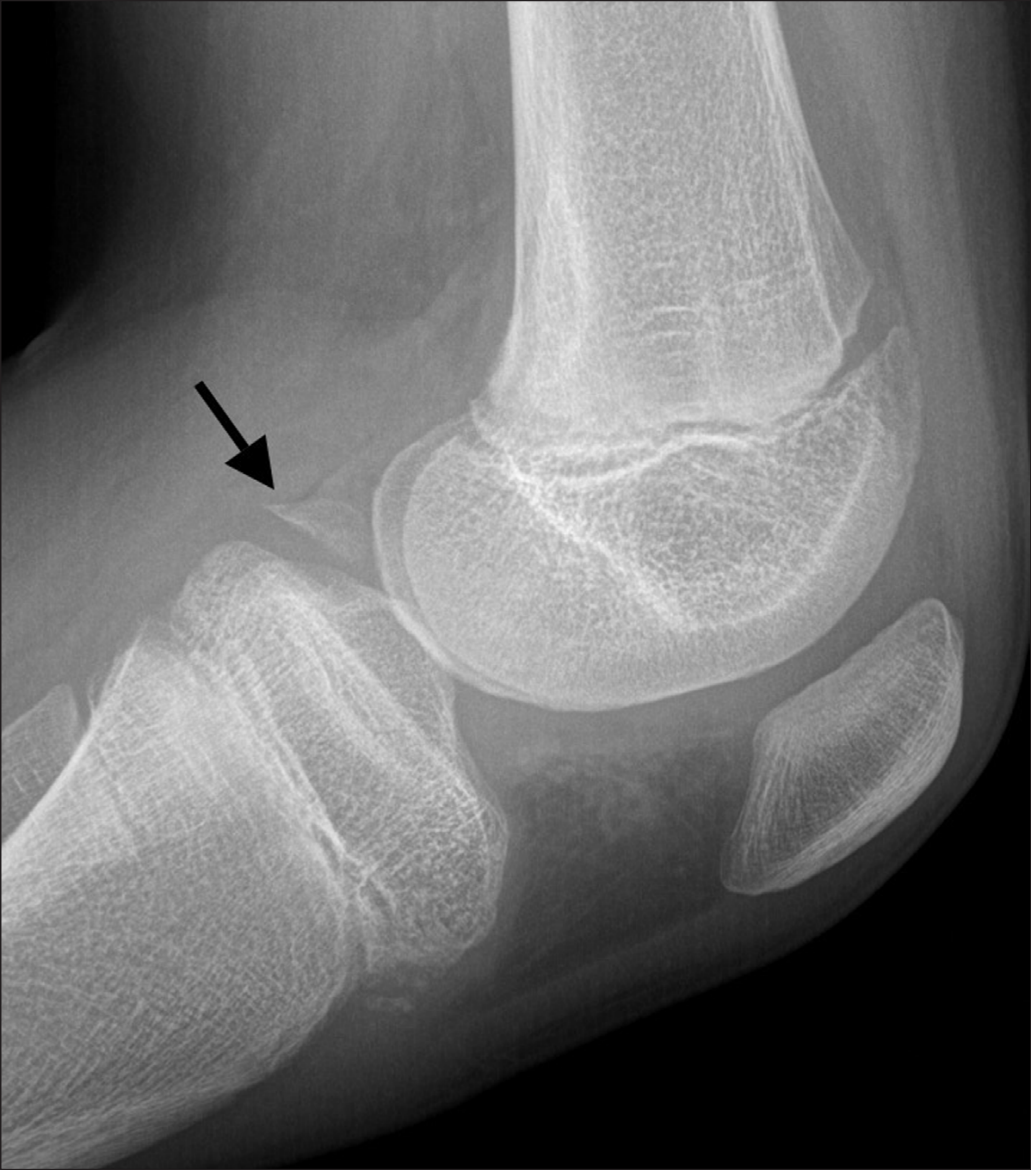


A 12-year-old boy with a known history of juvenile idiopathic arthritis (JIA) presented with pain in the left knee with associated joint effusion after a fall during ice-skating. Coronal fat-saturated (FS) proton density magnetic resonance imaging (MRI) revealed a hypointense structure at the intercondylar site of the posterior region of the medial meniscus (MM), which was initially interpreted as a meniscal fragment adjacent to a radial tear of the MM (Figure 1). Arthroscopy, however, was negative for a meniscal tear and demonstrated synovitis resulting from underlying JIA. MRI was repeated because of persistent symptoms and revealed a well-defined lesion within the fibrocartilage of the posterior horn of the MM. The center of the lesion was iso-intense to bone marrow with a peripheral hypointense rim on coronal T1-weighted images (WI) (Figure 2), indicative of a meniscal ossicle. In retrospect, this was not visible on sagittal T1-WI of the initial MRI. Radiograph confirmed a well-defined triangular ossification in the posteromedial joint space (Figure 3). JIA was conservatively treated.

## Comment

A meniscal ossicle is rare with a prevalence around 0.15% [1]. A meniscal ossicle consists of mature lamellar and cancellous bone surrounded by cortex and cartilage, embedded within the meniscus. The majority of ossicles are solitary and located in the posterior horn of the MM [1]. The etiology is still debated. Heterotopic ossification after single or repetitive trauma is often proposed as a pathogenetic mechanism. The presence of other intra-articular lesions in a large percentage of cases supports this theory, but conclusive arguments are still missing. Other theories include vestigial remnants, mucoïd degeneration, and tibial avulsion fracture of the meniscal root insertion [1].

Meniscal ossicle can be an incidental finding in asymptomatic patients, although most patients present with intermittent pain and joint effusion. Because of frequent coexisting intra-articular lesions, it is unclear whether these symptoms can be attributed to the meniscal ossicle.

Radiographs show a triangular or rectangular opacity usually at the posteromedial joint space, often misdiagnosed as a loose body. On MRI, location within the posterior horn of the MM differentiates it from a loose body, avulsion fracture of the semimembranosus tendon, and chondrocalcinosis. Furthermore, the ossicle appears isointense to bone marrow and is circumscribed by a hypointense rim on all sequences. The increased signal compared to the low signal of the fibrocartilage of the meniscus can mimic a tear. We highly recommend including T1-WI in at least one plane to the MRI protocol. This allows characterization of the fatty bone marrow content of the ossicle and to exclude chondrocalcinosis, which is of low signal on T1-WI. Because the causal relationship of the meniscal ossicle with the clinical presentation is not clear, the radiologist should scrutinize the knee for other abnormalities.

Conservative treatment of the meniscal ossicle is favoured. Arthroscopic resection may be considered in case of persistent symptoms and suspicion of posttraumatic etiopathogenesis of the meniscal ossicle [1].
